# *Aolatg1* and *Aolatg13* Regulate Autophagy and Play Different Roles in Conidiation, Trap Formation, and Pathogenicity in the Nematode-Trapping Fungus *Arthrobotrys oligospora*

**DOI:** 10.3389/fcimb.2021.824407

**Published:** 2022-01-25

**Authors:** Duanxu Zhou, Yingmei Zhu, Na Bai, Meihua Xie, Ke-Qin Zhang, Jinkui Yang

**Affiliations:** ^1^ State Key Laboratory for Conservation and Utilization of Bio-Resources, and Key Laboratory for Microbial Resources of the Ministry of Education, Yunnan University, Kunming, China; ^2^ School of Life Sciences, Yunnan University, Kunming, China

**Keywords:** *Arthrobotrys oligospora*, autophagy-related gene (*atg*), conidiation, trap formation, nematode predation, transcriptomic analysis

## Abstract

Autophagy is a conserved cellular recycling and trafficking pathway in eukaryotes that plays an important role in cell growth, development, and pathogenicity. Atg1 and Atg13 form the Atg1–Atg13 complex, which is essential for autophagy in yeast. Here, we characterized the roles of the *Aolatg1* and *Aolatg13* genes encoding these autophagy-related proteins in the nematode-trapping fungus *Arthrobotrys oligospora*. Investigation of the autophagy process by using the AoAtg8-GFP fusion protein showed that autophagosomes accumulated inside vacuoles in the wild-type (WT) *A. oligospora* strain, whereas in the two mutant strains with deletions of *Aolatg1* or *Aolatg13*, GFP signals were observed outside vacuoles. Similar results were observed by using transmission electron microscopy. Furthermore, deletion of *Aolatg1* caused severe defects in mycelial growth, conidiation, conidial germination, trap formation, and nematode predation. In addition, transcripts of several sporulation-related genes were significantly downregulated in the Δ*Aolatg1* mutant. In contrast, except for the altered resistance to several chemical stressors, no obvious differences were observed in phenotypic traits between the WT and Δ*Aolatg13* mutant strains. The gene ontology analysis of the transcription profiles of the WT and Δ*Aolatg1* mutant strains showed that the set of differentially expressed genes was highly enriched in genes relevant to membrane and cellular components. The Kyoto Encyclopedia of Genes and Genomes analysis indicated that differentially expressed genes were highly enriched in those related to metabolic pathways, autophagy and autophagy-related processes, including ubiquitin-mediated proteolysis and SNARE interaction in vesicular transport, which were enriched during trap formation. These results indicate that *Aolatg1* and *Aolatg13* play crucial roles in the autophagy process in *A. oligospora*. *Aolatg1* is also involved in the regulation of asexual growth, trap formation, and pathogenicity. Our results highlight the importance of *Aolatg1* in the growth and development of *A. oligospora*, and provide a basis for elucidating the role of autophagy in the trap formation and pathogenicity of nematode-trapping fungi.

## Introduction

Autophagy is a conserved degradation pathway that controls the homeostasis of the cellular environment by degrading organelles and proteins (Kroemer and Levine, 2008). Autophagy is induced in response to nutrient starvation and mediated by the cytoplasm-to-vacuole targeting (Cvt) pathway, which is responsible for specific sorting of proteins to vacuoles (Ying and Feng, 2019). Autophagy is tightly controlled by autophagy-related genes (*atg*), and approximately 42 Atg proteins have been identified in *Saccharomyces cerevisiae* (Zhu et al., 2019; Ying and Feng, 2019). Based on their specific functions, Atg proteins have been classified into six different groups: the Atg1 kinase complex, the Atg18–Atg2 complex, the phosphatidylinositol 3-kinase complex, the Atg12 conjugation system, the Atg8 conjugation system, and the Atg9 recycling complex (Nanji et al., 2017; Nakatogawa, 2020). The Atg1 complex is the initiator kinase complex for autophagy that serves as a scaffold to recruit downstream factors and regulate their functions *via* phosphorylation of serine or threonine residues (Nakatogawa, 2020). Atg1 is a serine/threonine protein kinase and the only enzymatic subunit of the Atg1 complex. Atg1 activity is essential for the turnover and recycling of other Atg proteins after the formation of autophagosomes (Cheong et al., 2008). Atg13 functions as a major positive regulator of Atg1 protein kinase and is highly phosphorylated under nutrient-rich conditions by protein kinase A and the target of rapamycin complex 1 (TORC1) (Kamada et al., 2000; Stephan et al., 2009). Under conditions of nutrient starvation or the presence of the specific inhibitor rapamycin, Atg13 is dephosphorylated, which allows its interaction with Atg1 and Atg17, resulting in the formation of the Atg1 complex and activation of the Atg1 kinase *via* its autophosphorylation (Memisoglu et al., 2019; Nakatogawa, 2020). In *S. cerevisiae*, the Atg1 complex consists of the protein kinase Atg1, the TORC1 substrate Atg13, and the trimeric Atg17–Atg31–Atg29 scaffolding subcomplex, which triggers autophagy when Atg1 and Atg13 assemble with the trimeric scaffold (Stjepanovic et al., 2014). The Atg1 complex mediates autophagosome formation by initiating phagophore assembly and localizing the downstream phosphatidylinositol 3-kinase complex, Atg9, and the ubiquitin-like conjugation systems to this membrane compartment (Chew et al., 2015).

In filamentous fungi, autophagy appears to be involved in nutrient recycling during starvation, and it has been suggested to regulate normal developmental processes. Several methods have been developed to visualize autophagy, such as transmission electron microscopy (TEM), GFP-Atg8 fusion protein, and probes for acidic compartment (Pollack et al., 2009). At present, the functions of Atg1 have been described in several filamentous fungi. For example, blockade of autophagy in the Δ*Mgatg1* mutant of *Magnaporthe oryzae* (syn. *Magnaporthe grisea*) impaired its ability to penetrate and infect the host (Liu et al., 2007). In *Aspergillus oryzae*, conidiation and development of aerial hyphae were suppressed in the Δ*Aoatg1* mutant, so AoAtg1 was deemed to be essential for nonselective autophagy and the Cvt pathway (Yanagisawa et al., 2013). Similarly, disruption of *Bbatg1* impaired autophagy, conidial yield, conidial germination, and virulence in *Beauveria bassiana* (Ying et al., 2016). Deletion of *Bcatg1* impaired autophagy and dramatically suppressed vegetative growth, conidiation, and sclerotium formation in the Δ*Bcatg1* mutant of *Botrytis cinerea* (Ren et al., 2017). Unlike Atg1, Atg13 has been characterized only in a limited number of fungi. In *M. oryzae*, the Δ*Moatg13* mutant displayed the phenotype similar to that of the wild-type (WT) strain (Dong et al., 2009), whereas in *A. oryzae*, the number of conidia was lower in the Δ*Aoatg13* mutant than in the WT strain (Kikuma and Kitamoto, 2011).

Nematode-trapping (NT) fungi are a specific filamentous group that can form unique mycelial structures (traps) for nematode predation, thus playing important roles in maintaining nematode population density in natural environments (Su et al., 2017). *Arthrobotrys oligospora* is a representative NT species that can live both saprophytically on organic matter and as a predator, by capturing tiny animals (Nordbring-Hertz et al., 2006). *A. oligospora* is used as the primary model for interactions between fungi and nematodes (Niu and Zhang, 2011). When stimulated by nematodes or other inducing factors, *A. oligospora* produces adhesive networks, indicating a switch from the saprophytic lifestyle to the predacious stage (Nordbring-Hertz, 2004; Yang et al., 2011). Since the sequencing of *A. oligospora* genome, an increasing number of studies has focused on the mechanism underlying trap formation, and several signaling proteins, such as regulators of G-protein, G-protein subunits and small GTPases, have been shown to regulate trap morphogenesis and lifestyle switch (Yang et al., 2018; Yang et al., 2020; Bai et al., 2021; Ma et al., 2021; Yang et al., 2021). Moreover, three orthologous Atg proteins have been identified in *A. oligospora*: deletion of *Aolatg8* blocked autophagy and abolished conidiation and trap formation (Chen et al., 2013), whereas deletion of *Aolatg4* and *Aolatg5* impaired autophagy and resulted in a reduction in conidia yields, cell nucleus number, and trap production (Zhou et al., 2020; Zhou et al., 2021). Despite their conserved features, autophagy proteins have different functions in fungi that are highly divergent morphology and lifestyle (Pollack et al., 2009; Ying and Feng, 2019).

In this study, to further probe biological impact of autophagy on the growth, development, and differentiation of NT fungi, the Atg1 and Atg13 homologs, which govern the first step of autophagy, were characterized in *A. oligospora* by gene disruption, phenotypic comparison, and transcriptomic analysis. Our results showed that Atg1 (AolAtg1) plays a crucial role in autophagy and underpins multiple phenotypic traits, whereas Atg13 (AolAtg13) plays a conserved role in autophagy and has a limited impact on the growth and development in *A. oligospora*. In addition, we compared transcriptional profiles of the WT and Δ*Aolatg1* mutant strains obtained using RNA-seq technology, which provided insights into the regulation of autophagy in *A. oligospora* and other NT fungi.

## Materials And Methods

### Strains and Media

The fungus *A. oligospora* (ATCC24927) and corresponding mutants were stored in the Microbial Library of the Germplasm Bank of wild species from Southwest China (Kunming, China). Potato dextrose agar (PDA), tryptone glucose (TG), and corn-maizena yeast extract (CMY) were prepared as described previously (Zhou et al., 2020; Zhou et al., 2021) and used to analyze fungal phenotypic traits. The complete medium (CM) and MM-N (0.5 g L^−1^ KCl, 0.5 g L^−1^ MgSO_4_, 1.5 g L^−1^ KH_2_PO_4_, 0.1% trace element, and 10 g L^−1^ glucose; pH 6.5) were used to compare colony growth and induce autophagy under nitrogen starvation (Talbot et al., 1993). Plasmids pRS426 and pCSN44 were maintained in the *Escherichia coli* strain DH5α (Takara, Shiga, Japan). *S. cerevisiae* (FY834) was used to screen the correctly recombined construct, and the selectionwas performed on the SC-Ura medium (Park et al., 2011). *Caenorhabditis elegans* (strain N2) was incubated on the oatmeal medium at 26°C for trap induction and bioassays.

### Sequence Analysis of AolAtg1 and AolAtg13 Proteins

The homologous sequences of AolAtg1 (AOL_s00076g234) and AolAtg13 (AOL_s00215g74) were retrieved from the *A. oligospora* genome (Yang et al., 2011) using the sequences of the orthologous proteins Atg1 (NP_011335) and Atg13 (NP_015511) in *S. cerevisiae*. The molecular mass and isoelectric point of the proteins were calculated using the online tool Compute pI/Mw (https://web.expasy.org/compute_pi/), and the conserved protein domains were predicted using InterPro (http://www.ebi.ac.uk/interpro/). The orthologs of AolAtg1 and AolAtg13 from other fungi were examined by BlastP, and the similarity between Atg1 or Atg13 homologs was analyzed using DNAman software (Version 6). A neighbor-joining tree was constructed using Mega software (7.0) (Kumar et al., 2016).

### Deletion of *Aolatg1* and *Aolatg13* Genes

The *atg* genes of *A. oligospora* were deleted using the homologous recombination method (Tunlid et al., 1999; Park et al., 2011). The upstream and downstream sequences corresponding to the genes *Aolatg1* and *Aolatg13* in *A. oligospora* were amplified using paired primers (**Supplementary Table S1**). Subsequently, the *hph* cassette for hygromycin resistance was amplified using primers Hph-f and Hph-r (**Supplementary Table S1**). Then, three PCR fragments and a linearized pRS426 vector were co-transformed into the yeast strain FY834 *via* electroporation. The complete fragment for gene disruption was amplified from the recombinant plasmid pRS426-Atg-hph using primers AolAtg1-5f/AolAtg1-3r or AolAtg13-5f/AolAtg13-3r (**Supplementary Table S1**), and it was transformed into *A. oligospora* using the protoplast transformation method as described previously (Tunlid et al., 1999; Liu et al., 2021; Long et al., 2021). The putative transformants were selected on the PDAS medium containing 200 g L^−1^ hygromycin B (Amresco, Solon, United States) (Liu et al., 2020; Xie et al., 2021). The successful deletions of the *Aolatg1* and *Aolatg13* genes were confirmed using PCR amplification and Southern blotting analyses, as described previously (Xie et al., 2019; Xie et al., 2020).

### Generation of the AoAtg8-GFP Fusion Protein

The pPK2-GFP-Sur (pPK2) vector harboring the green fluorescent protein (GFP) gene and the sulfonylurea resistance gene (*sur*) was used as a basic framework. The promoter fragment was amplified with primers AoP-f/AoP-r and inserted into the *Bsr*GI/*Spe*I sites of the pPK2 vector, and the cDNA fragment of *Aoatg8* (AOL_s00007g534) was amplified using the primer pair Atg8-f/Atg8-r (**Supplementary Table S1**) and then inserted into the *Bsr*GI/*Spe*I sites of the pPK2 vector. The resultant pPK2-GFP-AoAtg8 vector was inserted into the WT, Δ*Aolatg1*, and Δ*Aolatg13* mutant strains using the protoplast transformation method (Tunlid et al., 1999). The putative transformants were cultured on plates supplemented with 10 μg mL^−1^ chlorimuron ethyl, and GFP signals were examined under a confocal laser scanning microscope.

### Comparison of Mycelial Growth and Stress Resistance

The WT and mutant strains were incubated on PDA plates at 28°C for 5 days, then transferred onto PDA, CMY, TG, CM, and MM-N plates to evaluate their growth rate under different nutritional conditions, and the diameters of colonies were determined at 24 h intervals (Zhou et al., 2020; Zhou et al., 2021). To determine the levels of stress resistance, the fungal strains were incubated on TG plates supplemented with or without (control) different concentrations of chemical stressors, including oxidative agents (H_2_O_2_ and menadione) and osmotic agents (NaCl and sorbitol) at 28°C for 7 days. Relative growth inhibition (RGI) values of the fungal strains were calculated as previously described (Zhen et al., 2018). To compare the lipid droplets (LDs) of fungal mycelia, the WT and mutant strains were incubated on PDA plates for 5 days and then stained with 10 µg mL^−1^ BODIPY staining solution for 10 min. LDs were observed using a fluorescence microscope.

### Comparison of Conidiation and Transcription of Sporulation-Related Genes

To determine the spore yield, the WT and mutant strains were incubated on CMY plates at 28°C for 7 days, and then 5 mL of sterile water was added to each plate to harvest spores. Conidia were counted in 50 μL aliquots using a hemocytometer. To analyze spore germination, 50 μL suspensions (10^6^ spores per mL) of WT and mutant strains were added to the MM-N liquid medium at 28°C, and the number of germinated conidia was determined at 4, 8, and 12 h (Zhen et al., 2018).

To determine transcriptional levels of the sporulation-related genes, 50 μL conidial suspension aliquots (10^6^ spores per mL) of fungal strains were spread on CMY plates at 28°C. The fungal samples were harvested from the cultures grown for 3, 5, and 7 days and stored at −80°C for subsequent quantitative real-time PCR (qRT-PCR) analysis. The primers (**Supplementary Table S2**) for the target genes were designed using online software Primer3 (v0.4.0, https://bioinfo.ut.ee/primer3-0.4.0/). The expression of the *A. oligospora* β-tubulin gene (AOL_s00076g640) was used as the reference, and qRT-PCR analysis was performed as previously described (Yang et al., 2013). The transcript levels of each gene were analyzed using the 2^−ΔΔCt^ method (Livak and Schmittgen, 2001).

### Confocal Microscopy and TEM Assays

Hyphae of the WT and mutant strains were incubated in the CM medium at 28°C with gentle shaking at 180 r min^−1^ for 2 days, then transferred into the MM-N medium (nitrogen starvation) and incubated for 6 h. Hyphae were collected to observe autophagosome formation using TEM and confocal microscopy (Lv et al., 2017). The lipophilic styryl dye FM4-64 (Invitrogen, Carlsbad, CA, USA) was used for vacuole staining of hyphal cells, as described previously (Ma et al., 2020).

### Trap Induction and Bioassay

To induce trap formation, the conidia of fungal strains were collected from 7-day-old cultures on CMY plates, and 50 μL suspensions (10^6^ spores per mL) were incubated on water agar plates at 28°C for 3 days. Then, ~300 nematodes were added to each plate to induce trap formation, followed by microscopic observation of trap formation and nematode predation at 12 h intervals (Zhou et al., 2020).

### Transcriptomic Profile Analysis

To probe the mechanism by which AolAtg1 regulates autophagy, the WT and Δ*Aolatg1* mutant strains were incubated in the CMY medium at 28°C, and the spores were harvested 7 days post incubation. Next, 1×10^5^ spores were incubated on water agar plates at 28°C for 48 h, and the hyphae were harvested. Two treatment groups with three independent biological replicates were collected at 0 h without nematodes and following 24 h incubation after the addition of 300–400 nematodes. The hyphae were sent to the Shanghai Meiji Biological Company (Shanghai, China) for RNA sequencing and data analysis.

High-quality RNA samples were used to construct a sequencing library that was sequenced on an Illumina HiSeq 4000 system (Illumina, San Diego, CA, USA). The data were analyzed using the Majorbio Cloud Platform (www.majorbio.com). To identify differentially expressed genes (DEGs), transcripts per kilobase million (TPM) values were calculated for each gene and compared between the WT and Δ*Aolatg1* mutant strains. Gene abundance following RNA-seq was quantified by the expectation-maximization algorithm (Li and Dewey, 2011). Based on the quantitative expression results, DEGs were identified based on the following thresholds: | log2 ratio | ≥ 1 and adjusted *P* < 0.05. Gene Ontology (GO) and Kyoto Encyclopedia of Genes and Genomes (KEGG) analyses were performed to determine enrichment in GO terms of function classes and metabolic pathways in DEGs in comparison to the whole-transcriptome background. Sequence data were deposited in the National Center for Biotechnology Information (http://www.ncbi.nlm.nih.gov/) under the accession number PRJNA784322.

### Statistical Analysis

All experimental data are presented as the mean ± standard deviation (SD) of three biological replicates. Group effects were assessed by one-way analysis of variance followed by the Tukey’s honestly significant difference (HSD) test. Prism 5 (GraphPad, San Diego, CA, USA) was used to generate plots and perform statistical analyses. Effects were considered statistically significant if *P* < 0.05.

## Results

### Analysis of AolAtg1 and AolAtg13 Protein Sequences

The sequences of the Atg1 and Atg13 homologs were retrieved from the *A. oligospora* genome. AolAtg1 was found to consist of 949 amino acids with a predicted molecular mass and isoelectric point of 103.6 kD and 8.83, respectively. AolAtg13 was shown to comprise 984 amino acids with a predicted molecular mass and isoelectric point of 105.8 kD and 9.43, respectively. AolAtg1 contains a protein kinase domain (IPR000719) at the N-terminal and a serine/threonine-protein kinase at the C-terminal (IPR022708). AolAtg13 contains the autophagy-related protein 13 N-terminal domain (IPR018731). AolAtg1 shares a highly conserved protein sequence with homologous proteins from various NT fungi, having 94.2% and 86.8% identity to orthologous Atg1 from *Arthrobotrys flagrans* (syn. *Duddingtonia flagrans*) and *Dactylellina haptotyla*, respectively. AolAtg1 has moderate similarity (42.4–48.6%) to orthologs from different filamentous fungi and 32.4% identity with Atg1 of *S. cerevisiae* (**Supplementary Table S3**). Relative to AolAtg1, AolAtg13 also has a high degree of identity (72.7–91.2%) to orthologs from NT fungi, whereas its identity to orthologs from other filamentous fungi is low (23.2–29.5%), and it has only 10.6% identity with Atg13 from *S. cerevisiae* (**Supplementary Table S3**). Phylogenetic analysis showed that orthologous Atg1 and Atg13 from filamentous fungi were divided into two clades, whereas orthologous Atg1 or Atg 13 from NT fungi were clustered together (**Supplementary Figure S1**).

### *Aolatg1* and *Aolatg13* Are Involved in Mycelial Growth and Lipid Metabolism

**Figure 1:**
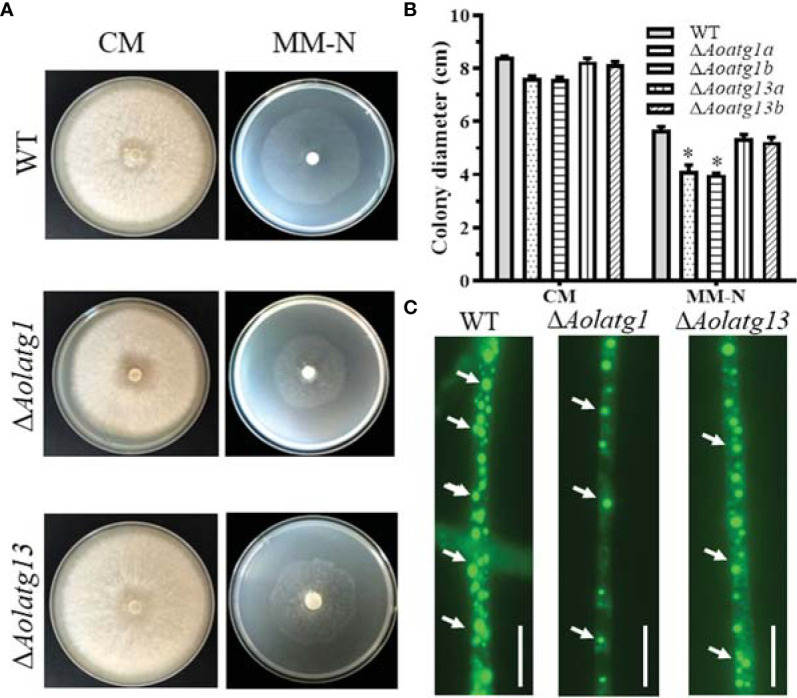
Comparison of mycelial growth in the WT and mutant strains of *Arthrobotrys oligospora* on CM and MM-N plates. **(A)** Colony morphology of fungal strains incubated on CM and MM-N plates at 28°C for 6 days. **(B)** Colony diameters of fungal strains incubated on CM and MM-N plates at 28°C for 6 days. Data are presented as the mean ± standard deviation. Statistical significance of differences between mutant strains and WT strain is indicated as follows: *P < 0.05 (Tukey’s HSD). **(C)** Comparison of lipid droplets (LDs) in the WT and mutant strains. The arrows indicate LDs stained with BODIPY staining solution. Scale bars = 10 µm.

Two independent positive transformants for *Aolatg1* and *Aolatg13* were screened and confirmed (**Supplementary Figure S2**), and their growth in various media was observed. The Δ*Aolatg1* mutant displayed lower hyphal growth on CMY, TG, and PDA plates. The colony diameter of the WT strain was 7.75 ± 0.25 cm on CMY plates at day 6, whereas those of the Δ*Aolatg1* and Δ*Aolatg13* mutants were 6.61 ± 0.26 cm and 6.94 ± 0.30 cm, respectively. Similarly, hyphal growth of the Δ*Aolatg1* mutant was lower than that of the WT strain in the CM and MM-N plates (**Supplementary Figure S3**; **Figures 1A, B**). After staining with BODIPY staining solution, the LDs in hyphal cells were visualized. The hyphal cells of the WT strain contained numerous LDs, whereas in Δ*Aolatg1* and Δ*Aolatg13* mutants displayed remarkably fewer LDs (**Figure 1C**).

### *Aolatg1* Regulates Sporulation and Spore Germination

**Figure 2:**
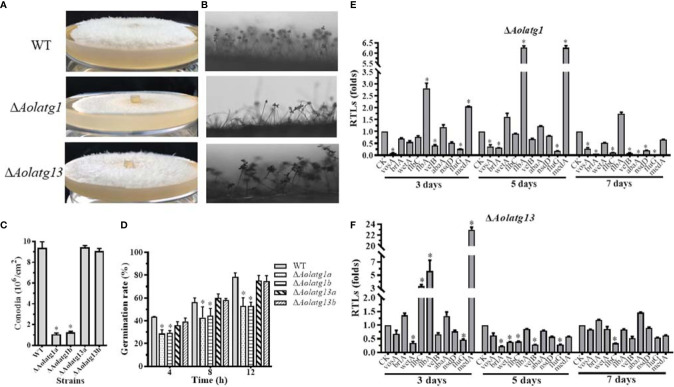
Comparison of conidiation and transcript levels of sporulation-related genes in the WT and mutant strains of *A. oligospora*. **(A)** Observation of aerial hyphae in the WT and mutant strains. **(B)** Observation of conidiophores in the WT and mutant strains. **(C)** Comparison of conidia yields in the WT and mutant strains. **(D)** Comparison of the germination rate in the WT and mutant strains. **(E)** The relative transcription levels (RTLs) of the sporulation-related genes in the WT and Δ*AolATG1* mutant strain. **(F)** The RTLs of sporulation-related genes in the WT and Δ*AolATG13* mutant strain. RTLs of the sporulation-related genes were assessed by comparing the levels of their transcription in the mutant strain with that of the WT strain. CK is the standard used in statistical analysis of the RTL of each gene in the deletion mutant compared to that in the WT strain under any given condition. Data are presented as the mean ± standard deviation. Statistical significance of differences between mutant strains and WT strain is indicated as follows: *P < 0.05 (Tukey’s HSD).

*Aolatg1* deletion resulted in defective growth of aerial hyphae on CMY plates (**Figure 2A**), and the conidiophores of Δ*Aolatg1* mutants became sparse compared to their number in the WT strain (**Figure 2B**). Thus, the loss of *Aolatg1* caused a significant reduction in spore yield: the Δ*Aolatg1* mutant produced 1.16 × 10^6^ conidia cm^-2^, which was 87.6% lower compared to the yield in the WT strain (9.40 × 10^6^ conidia cm^-2^) (**Figure 2C**). Furthermore, *Aolatg1* deletion caused a reduction in the spore germination rate: 29.0%, 47.6%, and 53.0% of the Δ*Aolatg1* mutant spores germinated at 4, 8, and 12 h, respectively, whereas 43.5%, 56.4%, and 78.4% of the WT strain spores germinated at the same time points (**Figure 2D**). However, no obvious differences in the numbers of aerial hyphae and conidiophores, spore yield, and spore germination rate were observed between the WT strain and Δ*Aolatg13* mutant.

The transcript levels of ten sporulation-related genes were determined in the WT, Δ*Aolatg1*, and Δ*Aolatg13* mutant strains using qRT-PCR at different growth stages. The transcript levels of *flbA* and *medA* were remarkably upregulated in the Δ*Aolatg1* mutant on days 3 and 5, and the remaining eight analyzed genes, including *abaA*, *brlA*, *flbC*, *fluG*, *nsdD*, *velB*, *vosA*, and *wetA*, were downregulated on day 7 (**Figure 2E**). In the Δ*Aolatg13* mutant, *flbC*, *flbA*, and *medA* were significantly upregulated on day 3, five genes (*brlA*, *flbC*, *fluG*, *velB*, and *wetA*) were downregulated on day 5, and only one gene, *flbC* was downregulated on day 7 (**Figure 2F**).

### *Aolatg1* and *Aolatg13* Are Involved in Stress Resistance

**Figure 3:**
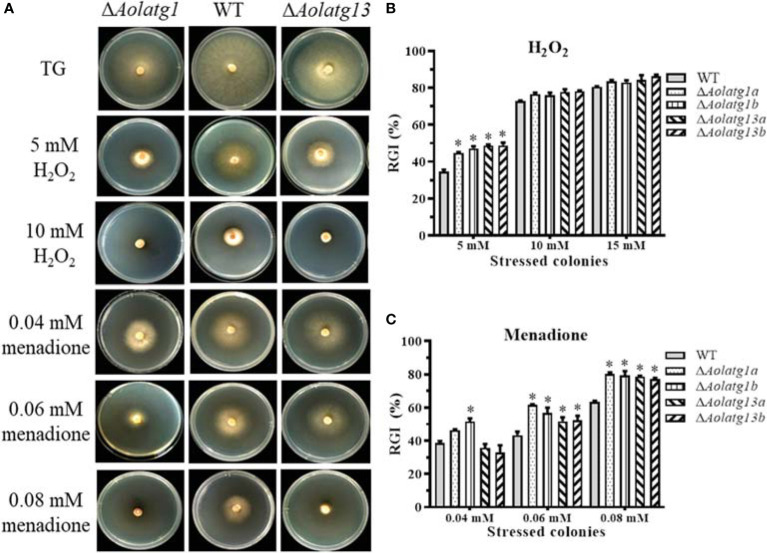
Comparison of tolerance to oxidative stress in the WT and mutant strains of *A. oligospora*. **(A)** Colonial morphology of the WT and mutant strains on TG plates or plates containing H_2_O_2_ and menadione at 28°C for 7 days. **(B)** Relative growth inhibition (RGI) of WT and mutant strains grown on the TG medium containing 5–15 mM H_2_O_2_ for 6 days. **(C)** RGI of WT and mutant strains grown on the TG medium containing 0.04–0.08 mM menadione for 6 days. Data are presented as the mean ± standard deviation. Statistical significance of differences between mutant strains and WT strain is indicated as follows: *P < 0.05 (Tukey’s HSD).

The stress response of fungal strains was evaluated on TG plates. We observed that deletion of *Aolatg1* and *Aolatg13* altered sensitivity to oxidative and osmotic agents. For example, deletion of *Aolatg1* and *Aolatg13* increased RGI by oxidative agents. In particular, in the presence of 5 mM H_2_O_2_, the RGI values of the Δ*Aolatg1* (45.9%) and Δ*Aolatg13* (48.7%) mutant strains were higher than that of the WT strain (34.5%), although no significant differences were noted at 10 and 15 mM H_2_O_2_ (**Figures 3A, B**). Further, the Δ*Aolatg1* and Δ*Aolatg13* mutants had higher RGI values in the presence of several menadione concentrations (0.04–0.08 mM and 0.06–0.08 mM, respectively) compared to the RGI value of the WT strain (**Figures 3A, C**). In addition, deletion of *Aolatg1* and *Aolatg13* altered sensitivity to osmotic agents. The Δ*Aolatg13* mutant had a higher RGI value at 0.2–0.3 M NaCl compared to that of the WT strain, whereas the sensitivity of the Δ*Aolatg1* mutant to NaCl was not changed significantly (**Supplementary Figure S4A, B**). Growth of both mutants was more strongly inhibited by sorbitol (0.5 M for Δ*Aolatg13* and 0.3 M for Δ*Aolatg1*) compared to the effect of sorbitol on the WT strain (**Supplementary Figure S4A, C**).

### *Aolatg1* and *Aolatg13* Regulate Autophagosome Formation

**Figure 4:**
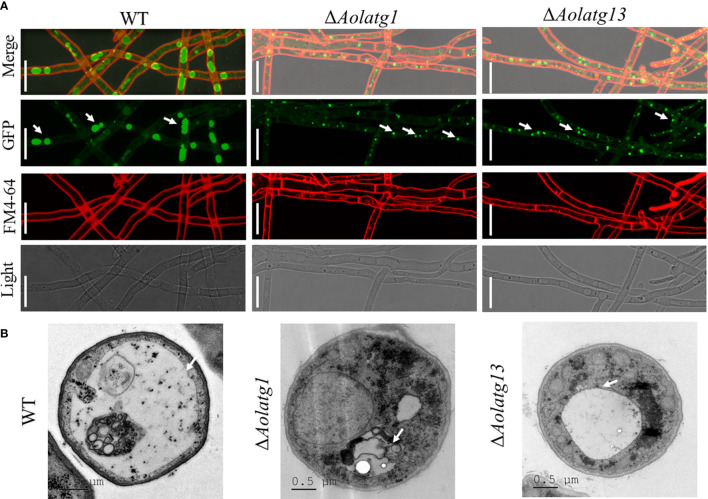
Comparison of the autophagy process in the WT and mutant strains of *A. oligospora*. **(A)** Expression of the GFP-Atg8 protein in the WT and mutant strains. The *A. oligospora* strains were grown in the liquid CM medium at 28°C for 24 h, and then transferred to the liquid MM-N medium for 6 h. The vacuoles were stained by FM4-64 and examined by fluorescence microscopy. White arrow: GFP signals. Scale bars = 10 µm. **(B)** The vacuoles of hyphal cells were observed using transmission electron microscopy. Arrows indicate the vacuole. Scale bars = 0.5 µm.

To probe the effect of *Aolatg1* and *Aolatg13* deletion on autophagy, we constructed a GFP-Atg8 fusion protein and expressed it in the WT, Δ*Aolatg1*, and Δ*Aolatg13* mutant strains. The WT and mutant strains were cultured in the CM medium for 24 h, and then transferred to the MM-N medium and incubated for 6 h. GFP-Atg8 signals were observed in the vacuoles of the hyphae in the WT strain (**Figure 4A**), whereas punctate GFP signals were observed near the vacuoles in the Δ*Aolatg1* and Δ*Aolatg13* mutants. We then used TEM to visualize autophagosomes and confirmed that when fungal strains were cultured in the MM-N medium for 6 h, autophagosomes were clearly observed in the vacuoles of the WT strain, whereas there were few autophagosomes or autophagosome-like structures in the vacuoles of the Δ*Aolatg1* and Δ*Aolatg13* mutants (**Figure 4B**).

### *Aolatg1* Regulates Trap Formation and Pathogenicity

**Figure 5:**
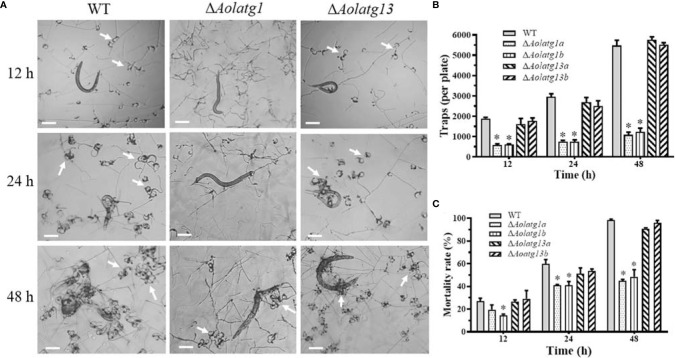
Comparison of trap formation and nematicidal activity in the WT and mutant strains of *A. oligospora*. **(A)** Traps (indicated by white arrows) were induced by nematodes at 12, 24, and 48 h. Scale bars = 100 µm. **(B)** Traps produced by the WT and mutant strains at 12, 24, and 48 h. **(C)** Nematode mortality rate (%) at 12, 24, and 48 h. Data are presented as the mean ± standard deviation. Statistical significance of differences between mutant strains and WT strain is indicated as follows: *P < 0.05 (Tukey’s HSD).

The WT and mutant strains were incubated on water agar plates at 28°C, followed by the addition of nematodes to induce trap formation. The WT and Δ*Aolatg13* mutant strains produced more traps than the Δ*Aolatg1* mutant (**Figure 5A**). At 12, 24, and 36 h, the WT strain produced 1,893, 2,953, and 3,804 traps per plate, respectively. The Δ*Aolatg13* mutant generated 1,606, 2,690, and 3,998 traps per plate at the same time points, whereas the Δ*Aolatg1* mutant produced only 582, 756, and 971 traps, respectively (**Figure 5B**). Accordingly, upon the formation of traps, 27%, 59.7%, and 99.5% of nematodes were captured by the WT strain at 12, 24, and 48 h, respectively, and for the Δ*Aolatg13* mutant the corresponding fractions were similar: 26.7%, 56.7%, and 90.9%. In contrast, only 19.5%, 41%, and 50% nematodes were captured by the Δ*Aolatg1* mutant at the corresponding time points (**Figure 5C**).

### Transcriptomic Analysis of the WT and Δ*Aolatg1* Mutant Strains

The transcriptomic profiles of the WT and Δ*Aolatg1* mutant strains were compared by RNA-seq. The raw and clean RNA-seq reads are shown in **Supplementary Table S4**. The percentage of phred-like quality scores at the Q30 level ranged from 91.5% to 92.9%, and the GC content ranged from 47.7% to 50.3% (**Supplementary Table S4**). The principal component analysis results showed that the WT and Δ*Aolatg1* mutant strains were located in different quadrants, suggesting that their transcription profiles were significantly different, whereas the independent samples at each time point were in close proximity, indicating high similarity and good reproducibility of the three repeats (**Supplementary Figure S4**).

**Figure 6:**
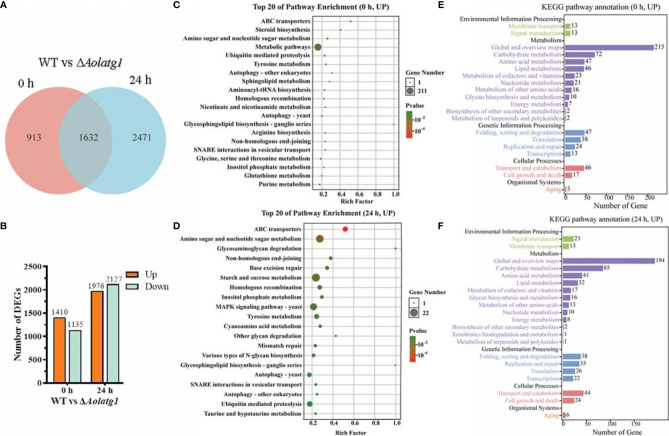
Comparison of differentially expressed genes (DEGs) between WT and the Δ*Aolatg1* mutant strain of *A. oligospora*. **(A)** Venn analysis of DEGs. **(B)** The number of upregulated and downregulated DEGs in the WT versus the Δ*Aolatg1* mutant strains. **(C)** The top 20 pathways that are upregulated in the WT strain compared to the Δ*Aolatg1* mutant strain at 0 h. **(D)** The top 20 pathways that are upregulated in the WT strain compared to the Δ*Aolatg1* mutant strain at 24 h. **(E)** Annotation of the upregulated KEGG pathways in the WT strain compared to the pathways in Δ*Aolatg1* mutant at 0 h. **(F)** Annotation of the upregulated KEGG pathways in the WT strain compared to the pathways in Δ*Aolatg1* mutant at 24 h.

A total of 2,545 and 4,103 DEGs were identified at 0 and 24 h, respectively, between the WT and Δ*Aolatg1* mutant strains, whereas expression levels of 1,632 genes were similar at these time points (**Figure 6A**). At 0 h, 1,410 genes were upregulated and 1,135 were downregulated in the WT compared to theΔ*Aolatg1* mutant (**Figure 6B**). The upregulated genes were enriched in 164 GO terms and 29 KEGG pathways (**Supplementary Figure S5B, C**). In particular, membrane (intrinsic/integral component of membrane, membrane part, and membrane), catalytic activity, and ion binding were the highly enriched terms in the GO analysis (**Supplementary Figure S6A**). In the KEGG analysis, the following metabolic pathways were highly enriched: carbohydrate metabolism, amino acid metabolism, and lipid metabolism (**Supplementary Figure S5C** and **Figure 6C**). Fold sorting and degradation, and translation were enriched in genetic information processing; transport and catabolism and cell growth and death were enriched in cellular processes (**Figure 6E**). The downregulated genes were enriched in 132 GO terms and 24 KEGG pathways (**Supplementary Figure S5B**). The catalytic activity was highly enriched in the GO analysis (**Supplementary Figure S6B**). In the KEGG analysis, metabolic pathways and biosynthesis of secondary metabolites were highly enriched, such as carbohydrate metabolism, amino acid metabolism, lipid metabolism, energy metabolism, nucleotide metabolism, and metabolism of terpenoids and polyketides (**Supplementary Figure S7**).

After induction with nematodes for 24 h, 1,976 genes were upregulated and 2,127 were downregulated in the WT strain compared to their levels in the Δ*Aolatg1* mutant (**Figure 6B**). The upregulated genes were enriched in 97 GO terms and 26 KEGG pathways (**Supplementary Figure S5B, C**), membranes were highly enriched in GO terms (**Supplementary Figure S6C**), whereas amino sugar and nucleotide sugar metabolism, starch and sucrose metabolism, MAPK signaling pathway, autophagy, ubiquitin-mediated proteolysis, and SNARE interaction in vesicular transport were enriched in KEGG pathways (**Figure 6D**), and carbohydrate metabolism, amino acid metabolism, and lipid metabolism were highly enriched in metabolism (**Figure 6F**). The downregulated genes were enriched in 198 GO terms and 28 KEGG pathways (**Supplementary Figure S5B, C**), cellular components were highly enriched in GO terms, such as the cytoplasm, ribosome, mitochondrion, and non-membrane-bounded organelle (**Supplementary Figure S6D**). In the KEGG pathway analysis, metabolic pathways and biosynthesis of secondary metabolites were enriched; with carbohydrate metabolism, amino acid metabolism, energy, and lipid metabolism being the particularly highly enriched processes (**Supplementary Figure S8**).

The comparison of the transcript levels of the genes associated with trap formation (**Table 1**) revealed that the expression of *flbA* was significantly increased by 5.69 folds in Δ*Aolatg1* mutant compared to the WT at 24 h post-induction (hpi) with nematode, and its expression was unaltered at 0 h. The expression of *hog1* in the Δ*Aolatg1* mutant was augmented by 1.92 folds at 24 hpi. However, the expression levels of *slt2* and *fus3* remained unperturbed in both WT and Δ*Aolatg1* mutant strains. The *atg8* expression level increased by 2.72 and 2.50 folds in the Δ*Aolatg1* mutant at 0 h and 24 hpi, respectively. Similarly, the expression of *ubr1* was elevated by 2.14 and 1.83 folds in the Δ*Aolatg1* mutant at 0 h and 24 hpi, respectively. Additionally, the expression levels of genes involved in oxidative stress response was also evaluated (**Table 1**). The comparative analysis demonstrated that the expression of genes *per* and *nox1* was enhanced by 2.80 and 3.71 folds, respectively in the Δ*Aolatg1* mutant compared to that in the WT at 24 hpi,. The expression of *cat2* was enriched by 4.15 and 12.84 folds in the Δ*Aolatg1* mutant at 0 h and 24 hpi, respectively. Similarly, the expression of *sod-2*, a gene encoding a superoxide dismutase was increased by 2.0 and 5.81 folds in the Δ*Aolatg1* mutant at 0 h and 24 hpi, respectively. In contrast, the expression of the *cat* gene (AOL_s00173g374) was decreased by 2.08 folds in the Δ*Aolatg1* mutant at 0 h. However, the expression of another *cat* gene (AOL_s00188g243) was reduced by 4.76 and 5.73 folds in the Δ*Aolatg1* mutant at 0 h and 24 hpi, respectively. In addition, the expression of *noxR* was also decreased by 2.54 folds in the Δ*Aolatg1* mutant at 24 hpi.

**Table 1:** Transcriptional response to *Aolatg1* deletion by the genes involved in trap formation and oxidative stress response in comparative transcriptome analysis.

Locus	Function annotation	Expressional levels				References
		TPM-0 h		TPM-24 h		
		WT	Δ*Aolatg1*	WT	Δ*Aolatg13*	
**Genes involved in trap formation and pathogenicity**						
AOL_s00215g516	*flbA*, developmental regulator	109.05	101.09	29.29	166.74	(Ma et al., 2021)
AOL_S00215g7	*ras2*, Ras family	480.70	601.97	130.02	75.16	(Yang et al., 2021)
AOL_s00054g446	*rab-7A*, Rab small GTPase	221.4	241.79	186.05	263.07	(Yang et al., 2018)
AOL_s00054g68	*glo3*, Arf GAP	119.8	138	100.8	127.71	(Ma et al., 2020)
AOL_s00110g154	*fus3*, MAP kinase	89.59	86.69	119.39	109.09	(Chen et al., 2021)
AOL_s00109g23	*hog1*, MAP kinase	146.71	148.25	137.99	265.83	(Kuo et al., 2020)
AOL_s00173g235	*slt2*, MAP kinase	169.76	150.98	191.17	186.58	(Zhen et al., 2018)
AOL_s00083g25	*stuA*, APSES transcription factor	51.04	37.8	48.2	45.78	(Xie et al., 2019)
AOL_s00007g534	*atg8*, autophagy-related protein 8	769.28	2096.53	596.99	1493.93	(Chen et al., 2013)
AOL_s00112g56	*hex1*, woronin body major protein	1590.13	2597.71	3231.56	2343.38	(Liang et al., 2017)
AOL_s00080g296	*ubr1*, E3 ubiquitin-protein ligase	40.98	87.82	25.94	47.47	(Zhang et al., 2021)
AOL_s00054g811	*velB*, developmental regulator	119.08	73.47	103.64	62.76	(Zhang et al., 2019)
**Genes involved in oxidative stress response**						
AOL_s00109g143	*per*, peroxidase	79.47	113.59	70.81	198.26	(Zhu et al., 2013)
AOL_s00173g374	*cat*, catalase	304.47	146.51	355.82	304.33	(Michán et al., 2003)
AOL_s00188g243	*cat*, catalase	1.19	0.25	1.49	0.26	(Wang et al., 2007)
AOL_s00006g411	*cat2*, catalase	1.48	6.14	3.00	38.53	(Sun et al., 2019)
AOL_s00193g69	*nox-1*, NADPH oxidase	120.89	178.27	67.51	250.78	(Li et al., 2017)
AOL_s00007g557	*nox-2*, NADPH oxidase	38.43	53.72	81.44	96.65	(Cano-Domínguez et al., 2008)
AOL_s00054g538	*noxR*, NADPH oxidase regulator	29.19	16.85	186.17	73.22	(Sun et al., 2019)
AOL_s00007g292	*sod*, superoxide dismutase	53.73	48.84	34.25	63.31	(Zhu et al., 2013)
AOL_s00054g687	*sodB*, superoxide dismutase	465.97	289.02	426.03	394.27	(Zhu et al., 2013)
AOL_s00170g93	*sod-2*, superoxide dismutase	603.01	1206.07	359.61	2089.07	(Zhu et al., 2013)

WT, wild-type strain; ΔAolatg1, Aolatg1 deletion mutant; -0 h, samples of the WT and ΔAolatg1 mutant strains in vegetative growth stage; -24 h, samples of the WT and ΔAolatg1 mutant strains after induced with nematodes for 24 h. Locus numbers and function were annotated according to the A. oligospora genome assembly (https://www.ncbi.nlm.nih.gov/). TPM, transcripts per kilobase million.

## Discussion

Autophagy is an evolutionarily conserved physiological process in eukaryotic cells that regulates programmed cell fate, tissue and cellular remodeling, and development (Pollack et al., 2009). Atg1 and Atg13 are core Atg proteins involved in the initial nucleation step of the phagophore formation (Itakura and Mizushima, 2010). Recently, Atg1 has been shown to be involved in the Cvt pathway and to play an essential role in the regulation of mycelial growth, conidiation, and virulence in filamentous fungi (Zhu et al., 2018; Ying and Feng, 2019). In this study, orthologs of Atg1 and Atg13 were characterized in the typical NT fungus *A. oligospora*, and their roles in autophagy, asexual development, trap formation, and nematode predation were comprehensively compared.

We used the GFP-Atg8 fusion protein to visualize autophagy and observed that in the WT strain, GFP signals accumulated in the vacuole, whereas in the Δ*Aolatg1* and Δ*Aolatg13* mutants, the majority of GFP signals were dispersed outside the vacuole in the hyphae, suggesting that the absence of the *Aolatg1* and *Aolatg13* genes blocked the autophagy pathway. Similar results have been reported for other fungi. For example, autophagic bodies were observed in the vacuoles of the WT strain of *Fusarium graminearum*, whereas no autophagic bodies or a small number of autophagosome-like structures were observed in the vacuoles of a Δ*Fgatg1* mutant (Lv et al., 2017). In *A. oryzae*, AoAtg1 is essential for nonselective autophagy and the Cvt pathway (Yanagisawa et al., 2013), and only a slight accumulation of EGFP-AoAtg8 in the vacuoles of the Δ*Aoatg13* mutant was observed (Kikuma and Kitamoto, 2011). Deletion of *Atg1* abolished autophagosome accumulation in the vacuoles of carbon-starved *Ustilago maydis* cells (Nadal and Gold, 2010). In addition, deletion of *Bcatg1* inhibited autophagosome accumulation in the vacuoles of nitrogen-starved *B. cinerea* cells (Ren et al., 2017). Similarly, autophagy was blocked in the Δ*Mgatg1* (Liu et al., 2007) and Δ*Bbatg1* mutants (Ying et al., 2016). These findings suggest that orthologs of Atg1 and Atg13 are indispensable for autophagy in various fungi.

Mycelial growth was impaired in the absence of *Aolatg1* and *Aolatg13* compared to that in the WT strain. The colony size and aerial mycelia of the Δ*Aolatg1* mutant were remarkably lower, whereas the mycelial growth of the Δ*Aolatg13* mutant was slightly reduced on the PDA, TG, and CMY media, but not on the CM and MM-N media. Moreover, deletion of *Aolatg1* and *Aolatg13* caused a remarkable reduction in LDs in hyphal cells. In *A. oryzae*, deletion of *Aoatg1* and *Aoatg13* did not affect colony size, but developed aerial hyphae were scarcely observed in the Δ*Aoatg1* mutant (Yanagisawa et al., 2013). Deletion of *Fgatg1* reduced the hyphal growth of *F. graminearum* (Lv et al., 2017), but colonies of the Δ*Fgatg13* mutant were the same as those of the WT strain when cultured on PDA plates (Lv et al., 2017). In *B. cinerea*, the mycelial radial growth rate of the Δ*Bcatg1* mutant was broadly similar to that of the WT, but the former produced significantly fewer aerial hyphae with more of them being fused (Ren et al., 2017). As in the Δ*Bcatg1* mutant of *B. cinerea*, the Δ*Mgatg1* mutant had sparse aerial hyphae on both CM and MM plates, although growth of its colonies was not affected (Liu et al., 2007). These findings suggest that orthologs of Atg1 play an essential role in mycelial development, especially in the growth of aerial hyphae, whereas Atg13 has only a minor role in these processes.

Deletion of *Aolatg1* caused a remarkable reduction in spore yield and spore germination rate, whereas deletion of *Aolatg13* did not affect the sporulation of *A. oligospora*. Transcription of several sporulation-related genes, such as *fluG*, *abaA*, *brlA*, and *velB*, was significantly downregulated in the Δ*Aolatg1* mutant compared to the WT strain on day 7, whereas in the Δ*Aolatg13* mutant, transcription levels of these genes showed no obvious change. Similarly, deletion of *Aoatg1* strongly inhibited the conidiation in *A. oryzae* (Yanagisawa et al., 2013), and the number of conidia was lower in the Δ*Aoatg13* mutant than in the WT strain (Kikuma and Kitamoto, 2011). In *B. cinerea*, the Δ*Bcatg1* mutant produced significantly fewer conidia than the WT strain, and most conidia showed an aberrant shape with many vacuoles (Ren et al., 2017). In *F. graminearum*, the conidiation was significantly reduced in the Δ*Fgatg1* and Δ*Fgatg13* mutants (Lv et al., 2017). Conidiogenesis in the Δ*Mgatg1* mutant was reduced significantly, and the conidia of Δ*Mgatg1* mutants germinated more slowly than those of the WT strain (Liu et al., 2007). Moreover, a mutant with disrupted *Bbatg1* had impaired conidial yield and conidial germination under starvation stress (Ying et al., 2016). These findings suggest that Atg1 plays a conserved and important role in the conidiation of many filamentous fungi, whereas the role of Atg13 in conidiation varies among fungi.

Apart from mycelial growth and conidiation, deletion of *Aolatg1* and *Aolatg13* impaired stress resistance, as the Δ*Aolatg1* and Δ*Aolatg13* mutants were more sensitive to oxidative stress caused by menadione and H_2_O_2_ than the WT strain. Furthermore, the Δ*Aolatg13* mutant was also sensitive to osmotic pressure (NaCl and sorbitol). It has been shown that deletion of *Aolatg4* and *Aolatg5* in *A. oligospora* also altered sensitivity to oxidative and osmotic stresses (Zhou et al., 2020; Zhou et al., 2021). In *B. bassiana*, the Δ*Bbatg8* mutant exhibited enhanced sensitivity to oxidative stress, whereas the Δ*Bbatg1* mutant did not (Ying et al., 2016). These results show that the autophagy pathway is also involved in regulating the fungal stress response.

Several studies have established that the asexual development of fungi is correlated to oxidative stress response. This is validated by the observation that Δ*cat-3* mutant of *Neurospora crassa* produces six times more aerial hyphae and conidia compared to the WT strain (Michán et al., 2003). Moreover, the depletion of *cat-1* resulted in a significant reduction in the rate of conidial germination (Wang et al., 2007). Recently, a Zn(II)2Cys6-type transcription factor, ADA-6 was identified in *N. crassa*. Characterization of *ada-6* revealed that its deletion impaired conidial production and induced female sterility. In addition, RNA-seq analysis demonstrated that ADA-6 modulates the transcription of *cat-3* and other genes participating in the production of reactive oxygen species during conidiation (Sun et al., 2019). In this study we found that the expressions of several genes associated with oxidative stress response were altered. The *cat-2* and *sod-2* genes were significantly enhanced in the Δ*Aolatg1* mutant compared to the WT strain at 0 and 24 hpi. However, *noxR* and the other two *cat* genes were downregulated in the Δ*Aolatg1* mutant. These findings suggest that defect in conidiation of Δ*Aolatg1* mutant might be connected to the oxidative stress response.

Trap formation in NT fungi is a complex cellular process that was suppressed by deletion of Atg4, Atg5, and Atg8 orthologs, which suggested that autophagy plays an important role in trap development in *A. oligospora* (Chen et al., 2013; Zhou et al., 2020; Zhou et al., 2021). In this study, we characterized the role of *Aolatg1* and *Aolatg13* in trap formation and nematode predation. Our results showed that trap formation and nematicidal activity were remarkably decreased in the Δ*Aolatg1* mutant. Autophagy has been recently demonstrated to be closely associated with fungal virulence. For example, deletion of *Mgatg1* caused lower turgor pressure of the appressorium, and the Δ*Mgatg1* mutant lost its ability to penetrate and infect host plants (Liu et al., 2007). However, the lack of *Mgatg13* did not have any influence on the pathogenicity of *M. oryzae* (Kershaw and Talbot, 2009). In *F. graminearum*, the pathogenicity of the Δ*Fgatg1* and Δ*Fgatg13* mutants was lower than that of the WT strain, as these mutants had decreased abilities to infect wheat spikelets and to spread to new spikelets following the original infection (Lv et al., 2017). In *B. cinerea*, most conidia of the Δ*Bcatg1* mutant lost the capacity to form the appressorium infection structure and failed to penetrate the onion epidermis, and pathogenicity assays showed that the virulence of Δ*Bcatg1* tested in different host plant tissues was drastically impaired (Ren et al., 2017). Moreover, the virulence of the Δ*Bbatg1* mutant was considerably weaker than that of the WT strain, as indicated by lower infectivity in the topical and intrahemocoel injection assays (Ying et al., 2016). These findings suggest that Atg1 plays a conserved and crucial role in the virulence of many pathogenic fungi, whereas Atg13 effect on virulence is prominent only in few fungi (e.g., *F. graminearum*).

Transcriptomic analysis showed that more DEGs were identified after the fungi were induced with nematodes compared to the transcriptomic differences between intact fungi. This finding suggests that many genes were mobilized during trap formation. In the GO analysis, the upregulated genes were highly enriched in the membrane-related terms at 0 h and 24 h, indicating that membrane trafficking plays a crucial role in autophagy, mycelial growth, and trap formation. In turn, the downregulated genes were enriched in catalytic activity at 0 h and in cellular components at 24 h, including ribosome, mitochondrion, and organelles, suggesting that there are multiple organelles involved in trap formation. In the KEGG analysis, the upregulated genes were highly enriched in metabolic pathways at 0 h; in contrast, except for metabolism, MAPK signaling pathways, autophagy, and autophagy-related processes such as ubiquitin-mediated proteolysis and ANARE interaction in vesicular transport were also enriched at 24 h. These findings suggest that MAPK pathways and autophagy play an important role in trap formation in *A. oligospora*. In fact, several MAPK signaling proteins have been proved to regulate trap formation in *A. oligospora*, such as Slt2 (Zhen et al., 2018; Xie et al., 2021), Hog1 (Chen et al., 2021), and Ime2 (Xie et al., 2020). Autophagy-related proteins such as AolAtg4 (Zhou et al., 2020), AolAtg5 (Zhou et al., 2021), and AolAtg8 (Chen et al., 2013) have also been shown to influence trap formation in *A. oligospora*, and deletion of *AolAtg1* significantly reduced of the number of traps. The set of downregulated genes was highly enriched in genes relevant to metabolic pathways and biosynthesis of secondary metabolites at 0 h and 24 h. Moreover, genes involved in lipid metabolism were enriched at 0 h and 24 h, suggesting that autophagy may regulate lipid metabolism. In *M. oryzae*, deletion of *the Mgatg1* gene influenced the number of lipid bodies, and lipid storage in conidia of the Δ*Mgatg5* mutant was lower than in the WT strain (Liu et al., 2007; Lu et al., 2009). Similarly, LDs accumulation was significantly reduced in the conidia of Δ*Bcatg1*, but the glycerol content was increased in Δ*Bcatg1* mutant (Ren et al., 2017). In addition, DEGs at 0 h and 24 h were enriched in genes involved in energy metabolism. Energy is required for trap development, as evidenced by the fact that deletion of the malate synthase gene led to a defect in trap formation (Zhao et al., 2014). Moreover, DEGs at 0 h and 24 h were enriched in genes relevant to metabolism of terpenoids and polyketides, which are involved in the biosynthesis of arthrobotrisins, a special group of metabolites identified in *A. oligospora* and other NT fungi (Anderson et al., 1995; Wei et al., 2011). Recently, 6-methylsalicylic acid, an intermediate in the biosynthesis of arthrosporols produced by NT fungi, was found to be a morphogen for spatiotemporal control of trap formation and a chemoattractant that lured *C. elegans* into fungal colonies (Yu et al., 2021). Therefore, transcriptomic analysis provides a good basis for understanding the mechanisms of mycelial growth, development, and pathogenicity.

G-protein signaling plays an indispensable role in trap formation of *A. oligospora*. There are several evidences corroborating this hypothesis, such as deletion of *flbA*, which encodes a regulator of G-protein signaling, abrogates trap formation in *A. oligospora* (Ma et al., 2021). Moreover, *hog1* deletion caused a reduction in trap formation and predation efficiency in *A. oligospora* (Kuo et al., 2020). In this study, we observed that the transcripts of *flbA* and *hog1* were markedly upregulated in Δ*Aolatg1* mutant when induced with nematodes. In addition, the expressions of *atg8* and *ubr1* were upregulated in Δ*Aolatg1* mutant during the stages of vegetative growth and trap formation. Atg8 has been shown to be indispensable for trap formation in *A. oligospora* (Chen et al., 2013). Additionally, the Δ*ubr1* mutant of *A. oligospora* exhibits a substantial reduction in vegetative growth and trap formation (Zhang et al., 2021). Therefore, according to these observations, it is evident that AolAtg1 regulates trap formation and pathogenicity of *A. oligospora* by promoting G-protein signaling and regulating protein ubiquitination.

At present, orthologs of Atg1 have been identified in many different fungi and shown to play conserved and indispensable roles in autophagy, mycelial growth, conidiation, lipid metabolism, and pathogenicity. In contrast, Atg13 has been identified only in a limited number of fungal species, and although it plays an essential role in autophagy, its deletion seemed to influence few if any phenotypic traits in most fungi, with the notable exception of *F. graminearum*. Here, we characterized Atg1 and Atg13 in *A. oligospora*, a typical NT fungus, and showed that AolAtg1 and AolAtg13 play crucial roles in autophagy, whereas their contributions to mycelial growth, conidiation, trap formation, and nematode predation are different. However, the underlying molecular mechanisms by which AolAtg1 and AolAtg13 regulate diverse phenotypes need to be further investigated using detailed comparative analysis of the transcriptome and by various other methods. Meanwhile, because of the lack of available resistance markers for *A. oligospora*, we failed to construct a double deletion mutant of *Aolatg1* and *Aolatg13*, which could help to understand the interaction between these two proteins in this fungus. Nonetheless, our results provide a solid basis for further investigation of the roles and regulatory mechanisms of *atg* genes in the growth, development, and pathogenicity of NT fungi.

## Data Availability Statement

The datasets presented in this study can be found in online repositories. The names of the repository/repositories and accession number(s) can be found below: https://www.ncbi.nlm.nih.gov/genbank/, PRJNA784322.

## Author Contributions

JY and K-QZ conceived and designed the study. DZ and YZ conducted the experiments. DZ, YZ, NB, and MX analyzed the data. JY, DZ, and YZ wrote and revised the manuscript. All authors have read and approved the final manuscript.

## Funding

This study was supported by the grants from the National Natural Science Foundation of China (No. 31960556) and the Applied Basic Research Foundation of Yunnan Province (No. 202001BB050004).

## Conflict of Interest

The authors declare that the research was conducted in the absence of any commercial or financial relationships that could be construed as a potential conflict of interest.

## Publisher’s Note

All claims expressed in this article are solely those of the authors and do not necessarily represent those of their affiliated organizations, or those of the publisher, the editors and the reviewers. Any product that may be evaluated in this article, or claim that may be made by its manufacturer, is not guaranteed or endorsed by the publisher.
