# Supplementary material for: Aolatg1 and Aolatg13 Regulate Autophagy and Play Different Roles in Conidiation, Trap Formation, and Pathogenicity in the Nematode-Trapping Fungus Arthrobotrys oligospora
Source: Front Cell Infect Microbiol. 2022 Jan 25;11:824407. doi: 10.3389/fcimb.2021.824407 (PMC8821819; doi:10.3389/fcimb.2021.824407)
Supplement: Supplementary file 1 [file DataSheet_1.pdf]

Supplementary Materials

Figure S1. Phylogenetic tree analysis based on the amino acid sequences of Atg homologous proteins from different fungi. GenBank accession numbers are provided in brackets. The amino acid sequences of Atg proteins were aligned using ClustalX version 1.83, and MEGA 7 was used to construct a neighbor-joining tree, including bootstrap analysis with 1,000 replicates. A. Phylogenetic tree of Atg1 orthologs. B. Phylogenetic tree of Atg13 orthologs.

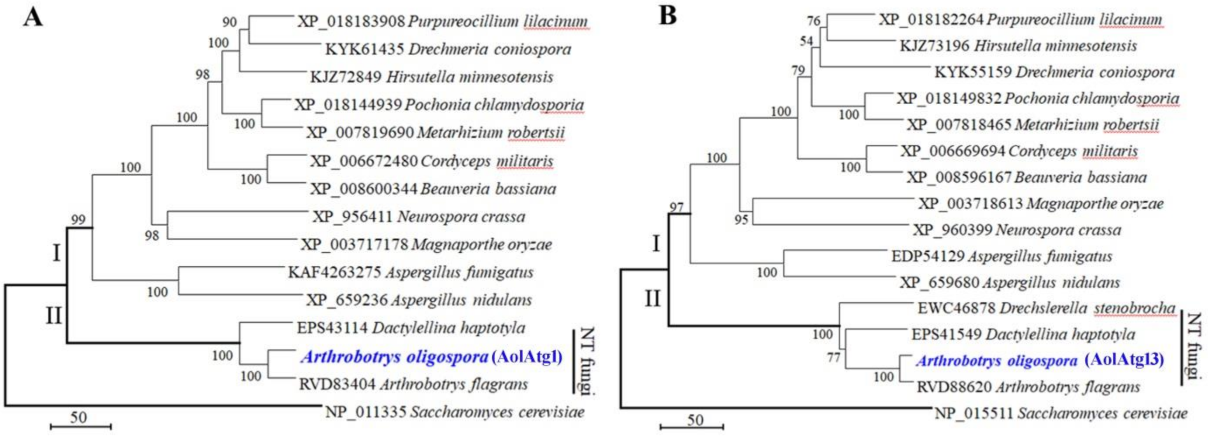

Figure S2. Schematics of gene knockouts and verification. A. *Aolatg1* knockout and verification. (a) Diagram of homologous recombination of *Aolatg1* and the homologous flanks of the target gene. (b) PCR confirmation of *Aolatg1* deletion in the transformants. (c) Southern blotting analysis of the WT strain and transformants. B. *Aolatg13* knockout and verification. (a) Diagram of homologous recombination of *Aolatg13* and the homologous flanks of the target gene. (b) PCR confirmation of *Aolatg13* deletion in the transformants. (c) Southern blotting analysis of the WT strain and transformants.

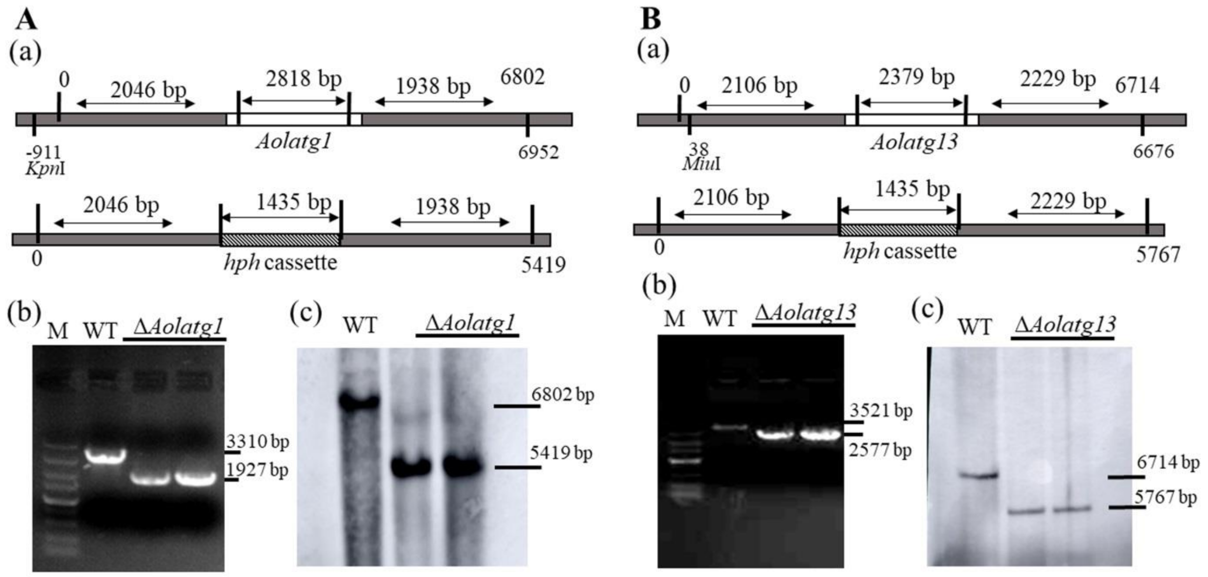

Figure S3. Comparison of colony morphology and mycelial growth in the WT and mutant strains of *A. oligospora*. (A) Colony morphology of the WT and mutant strains cultured on PDA, CMY, and TG plates for 6 days at 28 ℃. (B) Colony diameters of the WT and mutant strains incubated on PDA, CMY, and TG plates. Data are presented as the mean ± standard deviation from three replicates.

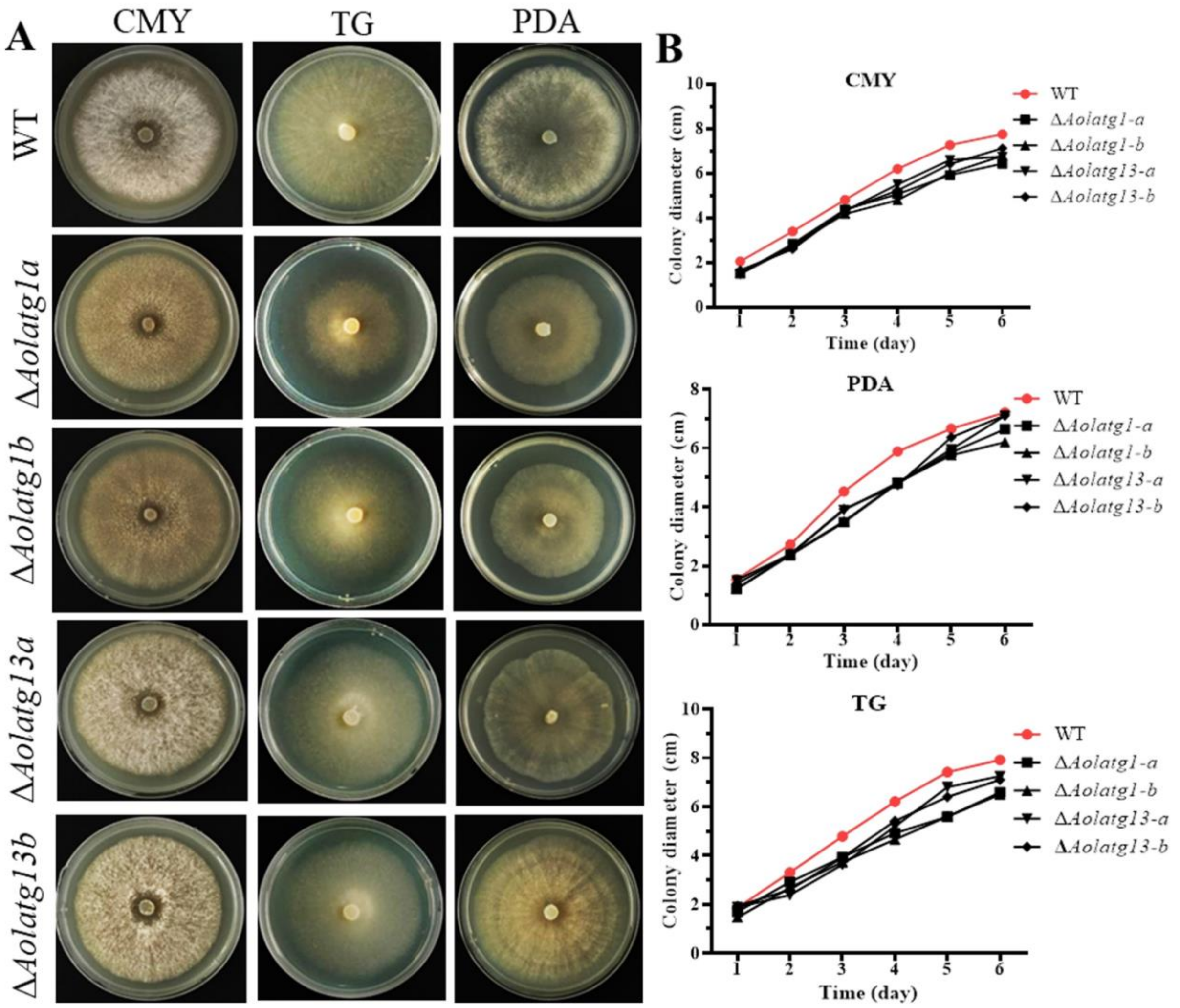

Figure S4. Comparison of tolerance to NaCl and sorbitol in the WT and mutant strains of *A. oligospora*. A. Colony morphology of the WT and mutant strains cultured on TG plates or plates containing NaCl and sorbitol. B. Relative growth inhibition (RGI) of the WT and mutant strains cultured on the TG medium containing 0.1–0.3 M NaCl for 6 days. C. RGI of the WT and mutant strains cultured on the TG medium containing 0.3–0.7 M sorbitol for 6 days. Data are presented as the mean ± standard deviation. Statistical significance of differences between mutant strains and WT strain is indicated as follows: $*P < 0.05$ (Tukey's HSD).

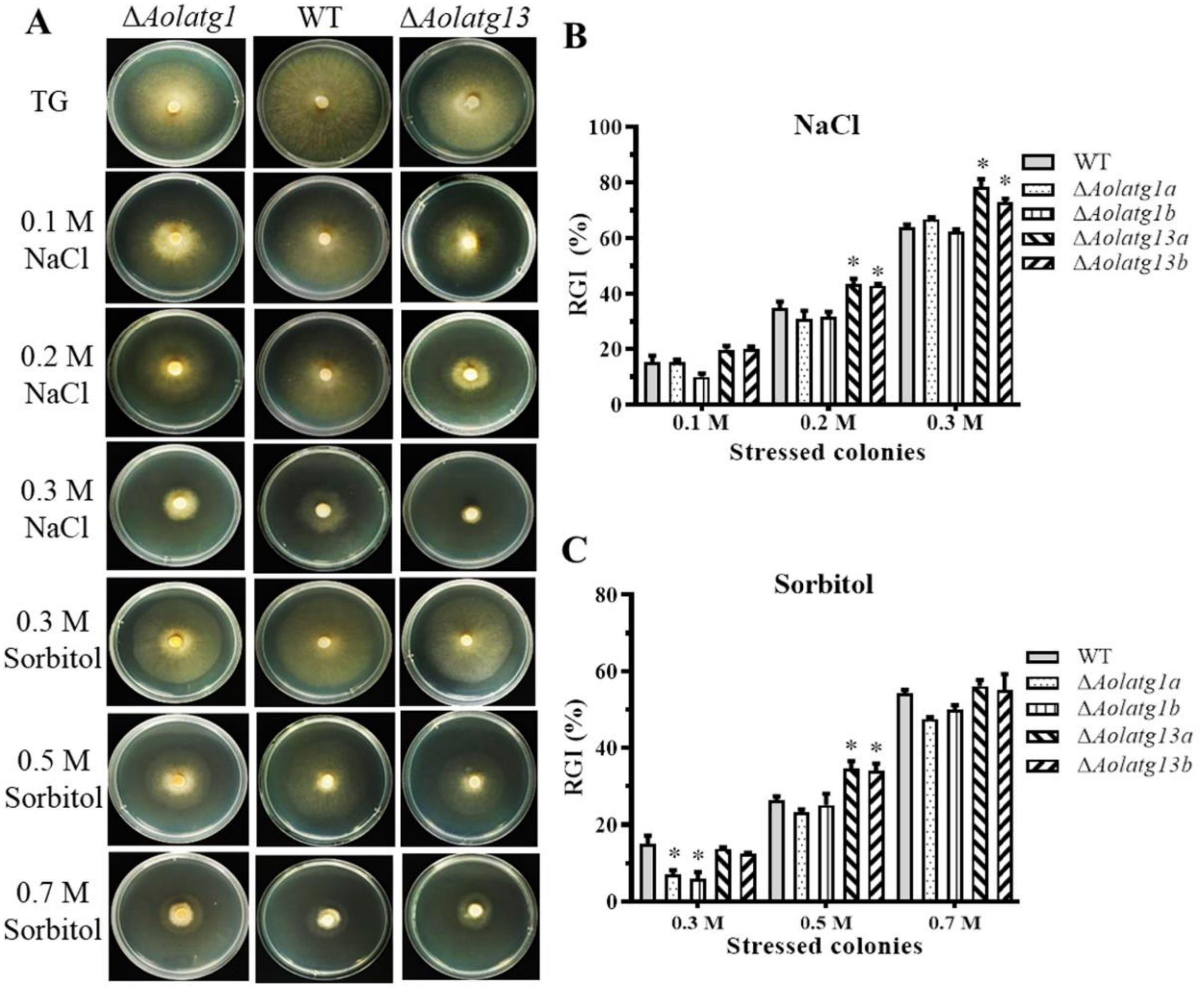

Figure S5. Principal component analysis (PCA), GO, and KEGG enrichments of the transcriptome. A. PCA analysis of the WT and Δ*Aolatg1* mutant transcriptomes. WT indicates the wild-type strain, and ATG1 indicates the Δ*Aolatg1* mutant. B. The number of upregulated and downregulated GO terms at 0 h and 24 h. C. The number of upregulated and downregulated KEGG pathways at 0 h and 24 h.

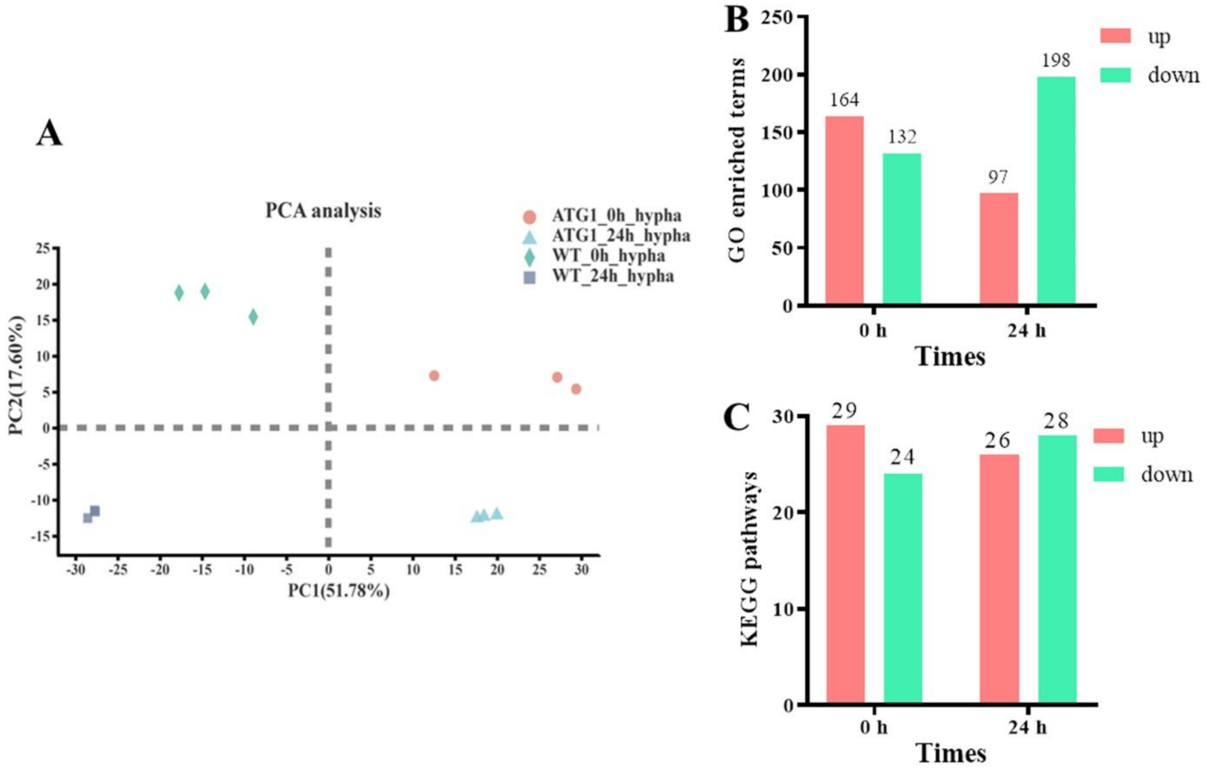

Figure S6. Gene Ontology (GO) enrichment of the differentially expressed genes (DEGs) in the WT and Δ*Aolatg1* mutant strains of *A. oligospora*. A. Top 20 of upregulated GO terms enrichment of the WT versus Δ*Aolatg1* mutant samples at 0 h. B. Top 20 of downregulated GO terms enrichment of the WT versus Δ*Aolatg1* mutant samples at 0 h. C. Top 20 of upregulated GO terms enrichment of the WT versus Δ*Aolatg1* mutant samples at 24 h. D. Top 20 of downregulated GO terms enrichment of the WT versusΔ*Aolatg1* mutant samples at 24 h.

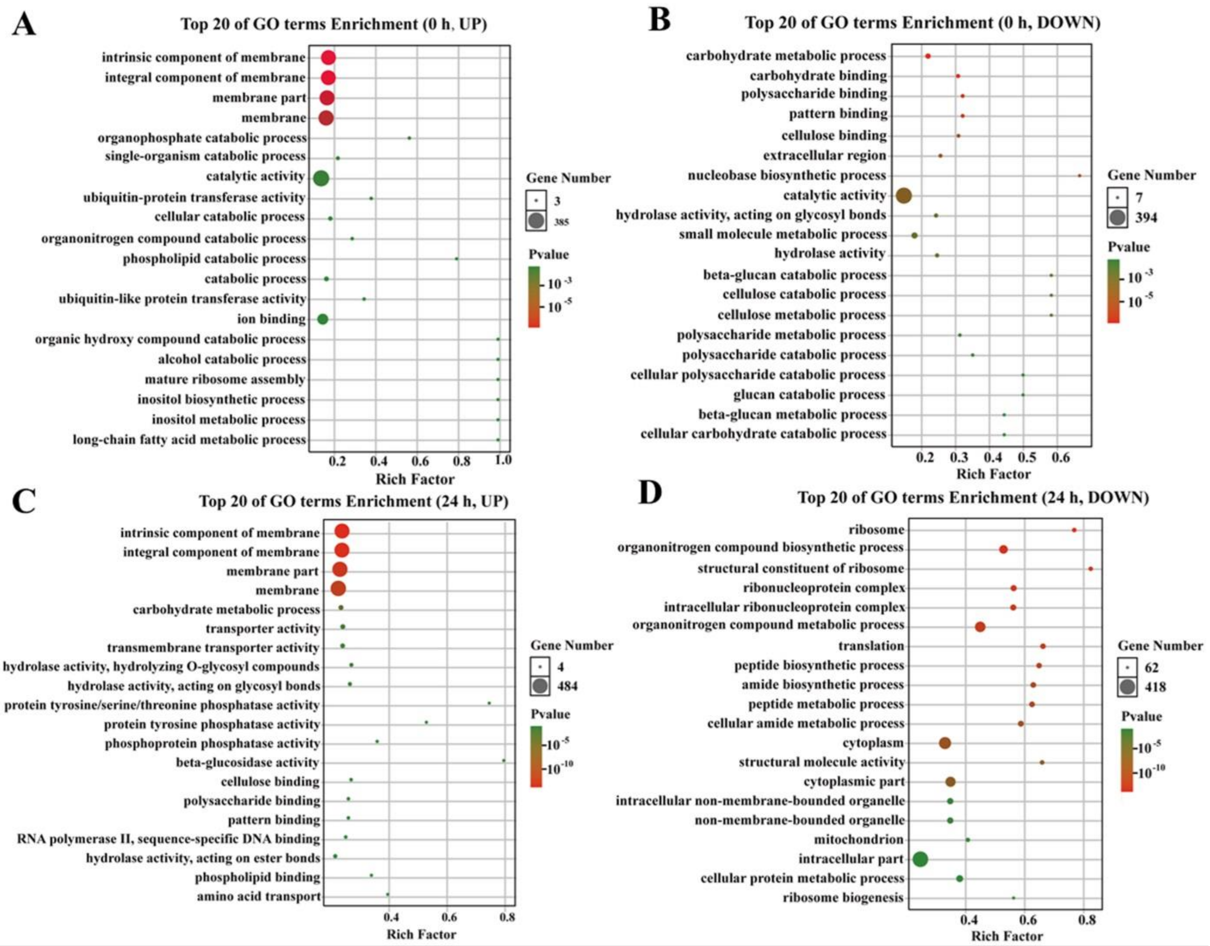

Figure S7. KEGG enrichment of the differentially expressed genes (DEGs) in the WT and Δ*Aolatg1* mutant strains of *A. oligospora* at 0 h. A. Top 20 pathways downregulated in the WT versus Δ*Aolatg1* mutant samples at 0 h. B. Annotation of KEGG pathways downregulated in the WT versus Δ*Aolatg1* mutant samples at 0 h.

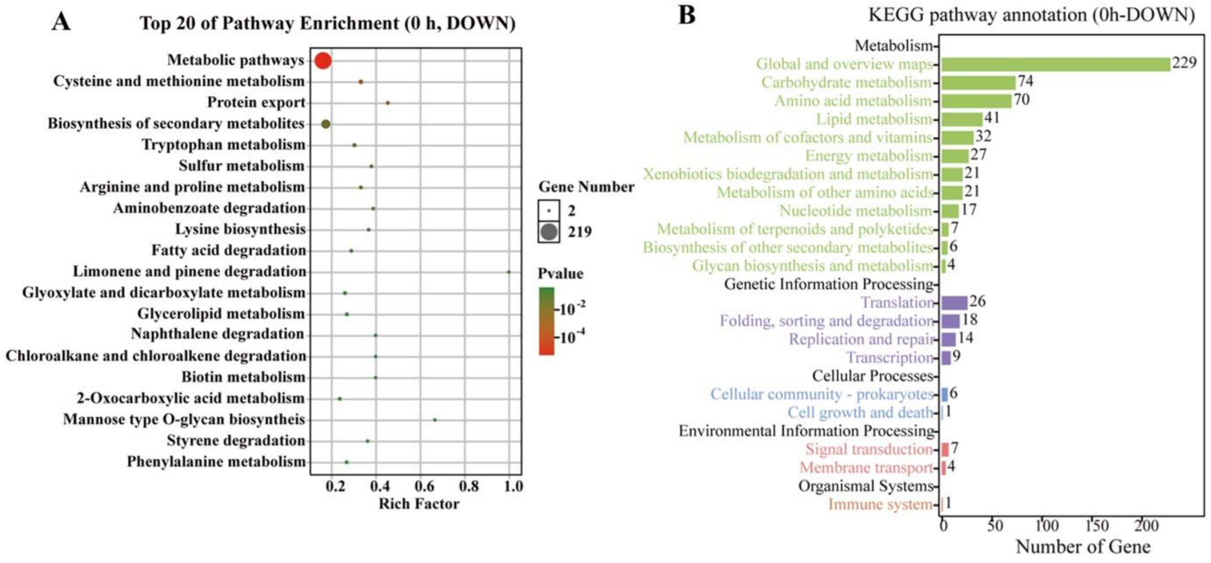

Figure S8. KEGG enrichment of the differentially expressed genes (DEGs) between the WT and Δ*Aolatg1* mutant strains of *A. oligospora* at 24 h. A. Top 20 pathways downregulated in the WT versus Δ*Aolatg1* mutant samples at 24 h. B. Annotation of KEGG pathways downregulated in the WT versus Δ*Aolatg1* mutant samples at 24 h.

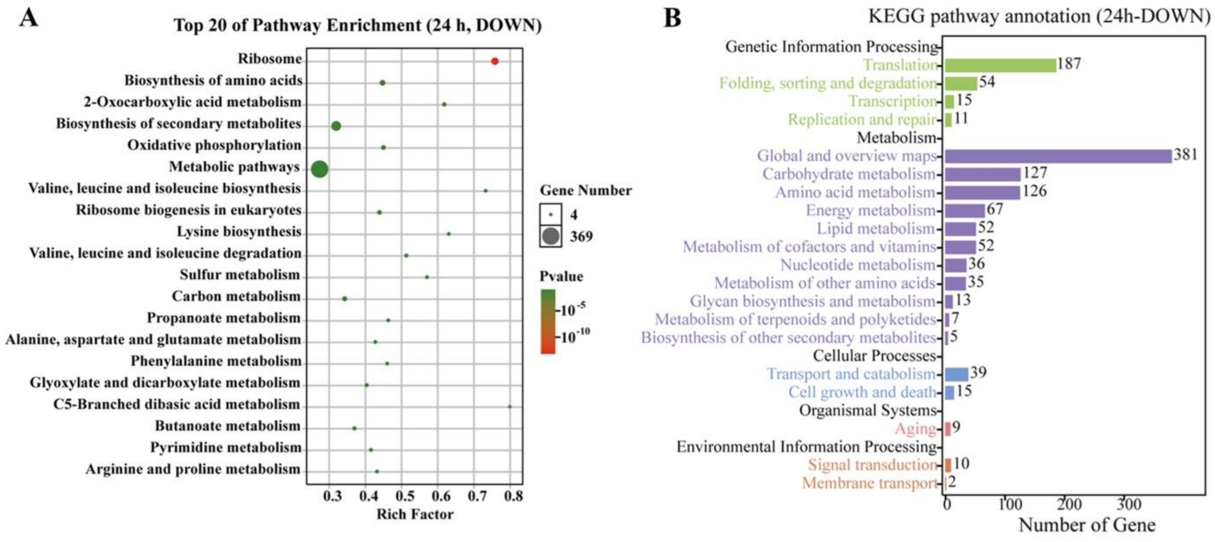

Table S1. List of primers used for gene manipulation in this study.

AolAtg1-5F	GTAACGCCAGGGTTTTCCCAGTCACGACGGAATCCTTTCTCCTGCTCTTTT	Amplify the *Aolatg1* gene
AolAtg1-5R	ATCCACTTAACGTTACTGAAATCTCCAACGCCTATTTCCTTGCCTATCCT	5’ flank
AolAtg1-3F	CTCCTTCAATATCATCTTCTGTCTCCGACTATTCTTGGTATCCGTGGGC	Amplify the *Aolatg1* gene
AolAtg1-3R	GCGGATAACAATTTCACACAGGAAACAGCTTTTGTTTCGTGATCTGTTTGT	3’ flank
AolAtg13-5F	GTAACGCCAGGGTTTTCCCAGTCACGACGAGGTGCAGGTTACGTGTATGA	Amplify the *Aolatg13*
AolAtg13-5R	ATCCACTTAACGTTACTGAAATCTCCAACCACTTCTACAACCGCCACA	gene 5’ flank
AolAtg13-3F	CTCCTTCAATATCATCTTCTGTCTCCGACAGTGATAGTCGGTGCTGGTG	Amplify the *Aolatg13*
AolAtg13-3R	GCGGATAACAATTTCACACAGGAAACAGCGACTGCGTACTATTGCCGTTA	gene 3’ flank
Hph-f	GTCGGAGACAGAAGATGATATTGAAGGAGC	Amplify the *hph* cassette
Hph-r	GTTGGAGATTTCAGTAACGTTAAGTGGAT	
YZ-ATG1-F	ATCCTTTCTCCTGCTCTTTTG	Verify the transformants
YZ-ATG1-R	CTCACAGTCACCCAGTTTATCC	
YZ-ATG13-F	GCAAGCACAACTATACAAACCC	Verify the transformants
YZ-ATG13-R	CCACCACTTTCCAGCGTTA	
Probe-ATG1-F	AACAATAGTAAACCGCACCAG	Make Southern blotting
Probe-ATG1-R	CTATGGGTTTTGCTTGGTGT	probe
Probe-ATG13-F	TCATTTCCCGTCTAATCCTG	Make Southern blotting
Probe-ATG13-R	TCGCCGAGTTGTATCCCT	probe
AoP-f	AGGAGGTCAAAGCTTACCAGATACGATTAATCCAGCATCTCACGACC	Amplify the promoter of
AoP-r	ATGGTGAGCAAGGGCGAGGAGCTTCATAGATTTGGGTGTCGGTT	*Aoatg8*
Atg8-f	GTACAGCTCGTCCATGCCGAGAGTGAATGGCACGATCAAAGTT	Amplify the cDNA of
Atg8-r	AAGTAAACAATCAATCCATTTCGCTATTCAGGCAAGGTAC	gene *Aoatg8*

Table S2. Primers used for RT-PCR analysis of the sporulation-related genes.

Description	Gene name	Sequence (5′-3′)
	AOL_s00169g18	18-5F-AAGCTACACCCAATCAACGC
	(*veA*)	18-3R-TTGCGATGCTGACGATCTTG
	AOL_s00007g157	157-5F-CTCTCCGGCAAAGACAATCG
	(*flbC*)	157-3R-GTCGACTGAGGATAGTAGCT
	AOL_s00075g211	211-5F-ATTACGGCCGCCTAGTAGTC
	(*nsdD*)	211-3R-CTCGTTTGGACCTGGTTGTG
	AOL_s00043g361	361-5F-GATTCCAGTCCCGTGAATTC
	(*fluG*)	361-3R-GCTAAGGAGAGGATGGGCAT
	AOL_s00006g570	570-5F-GCGGATCCAACATGAAGCTT
	(*rodA*)	570-3R-GGTTGACAACTGGGATGCTG
	AOL_s00080g63	63-5F-AACTTTATGCGCCTTGTCGT
**Sporulation-related**	(*abaA*)	63-3R-TTGGCTAGGTGGTCTGTACG
**genes**	AOL_s00054g700	700-5F-CAAACCACCCACCACCAAAT
	(*vosA*)	700-3R-GGATGGACAGGAGAAGGACC
	AOL_s00215g516	516-5F-TTCAAACGCAGCTCCTTCAC
	(*flbA*)	516-5R-AAGCGGGTTGACAGATGAGA
	AOL_s00210g120	120-5F-TCCGGCCCAATGATTCAGAA
	(*medA*)	120-5R-AGATCGCAGGAACATGGTGA

	AOL_s00097g514 (*brlA*)	514-5F-AACTCCATCACCATCCGTAA<br>514-5R-CAGGATATTCGGCACTCA
	AOL_s00173g221 (*wetA*)	221-5F-CCCTGTGCTACTATTGCTAC<br>221-5R-CCGTTGCGAGCATTTCTT
	AOL_s00054g811 (*velB*)	811-5F-ATTCCGCAACTTCTCCCTCA<br>811-3R-GGCATGTTTGGATTCTGGGG
**β-tubulin gene**	AOL_s00076g640 (*tub*)	tubA-F-CCACCTTCGTCGGTAACTC<br>tubA-R-TCGTCCATACCCTCACCAG

Table S3. Sequence similarity between AolATG1/AolATG13 and orthologs from different fungi. Sequence similarity of orthologous Atg was analyzed using DNAman software.

Species	Similalrity to AolAtg1 (%)	Similalrity to AolAtg13 (%)
Arthrobotrys oligospora	100	100
Arthrobotrys flagrans	94.2	91.2
Dactylellina cionopaga	86.8	72.7
Drechslerella stenobrocha	-	73.0
Aspergillus fumigatus	48.6	29.3
Aspergillus nidulans	43.7	29.5
Purpureocillium lilacinum	43.5	26.9
Neurospora crassa	42.4	24.4
Magnaporthe oryzae	43.4	23.2
Pochonia chlamydosporia	44.1	25.9
Hirsutella minnesotensis	45.5	26.4
Metarhizium robertsii	43.8	25.5
Drechmeria coniospora	44.2	25.4
Cordyceps militaris	44.8	26.0
Beauveria bassiana	45.0	25.0
Saccharomyces cerevisiae	32.4	10.6

Table S4. Sequencing data statistics from the transcriptome analysis.

Sample	Total reads	Total mapped	Multiple mapped	Uniquely mapped	Raw reads	Raw bases
ATG1_0h_1	52341788	50735512(96.93%)	191235(0.37%)	50544277(96.57%)	52946894	7994980994
ATG1_0h_2	52344310	50703965(96.87%)	188401(0.36%)	50515564(96.51%)	52808984	7974156584
ATG1_0h_3	53624344	51847772(96.69%)	197465(0.37%)	51650307(96.32%)	54200806	8184321706
ATG1_24h_1	51311728	30379398(59.21%)	97367(0.19%)	30282031(59.02%)	51670210	7802201710
ATG1_24h_2	56392234	25567803(45.34%)	79045(0.14%)	25488758(45.2%)	56840660	8582939660
ATG1_24h_3	55855454	27584228(49.39%)	85956(0.15%)	27498272(49.23%)	56392760	8515306760
WT_0h_1	46898482	45416135(96.84%)	183226(0.39%)	45232909(96.45%)	47509132	7173878932
WT_0h_2	46964880	45600127(97.09%)	183498(0.39%)	45416629(96.7%)	47471022	7168124322
WT_0h_3	50482982	48890863(96.85%)	192894(0.38%)	48697969(96.46%)	50953732	7694013532

WT_24h_1	51387924	30866871(60.07%)	101070(0.2%)	30765801(59.87%)	51783536	7819313936
WT_24h_2	49958832	28950733(57.95%)	89782(0.18%)	28860951(57.77%)	50356978	7603903678
WT_24h_3	49840398	25559605(51.28%)	74186(0.15%)	25485419(51.13%)	50263842	7589840142

**Sample**	**Clean reads**	**Clean bases**	**Error rate (%)**	**Q20 (%)**	**Q30 (%)**	**GC content(%)**
ATG1_0h_1	52341788	7760699009	0.0277	97.1	91.54	47.96
ATG1_0h_2	52344310	7776548505	0.0272	97.32	91.95	47.97
ATG1_0h_3	53624344	7974658396	0.0275	97.18	91.68	48.02
ATG1_24h_1	51311728	7643687922	0.0262	97.66	92.8	49.7
ATG1_24h_2	56392234	8371018602	0.0264	97.58	92.63	50.31
ATG1_24h_3	55855454	8310953513	0.0273	97.28	91.89	50.33
WT_0h_1	46898482	6938431303	0.0272	97.28	91.92	47.73
WT_0h_2	46964880	6978104784	0.027	97.37	92.09	47.97
WT_0h_3	50482982	7492817315	0.027	97.37	92.12	47.97
WT_24h_1	51387924	7668437697	0.0266	97.53	92.5	49.95
WT_24h_2	49958832	7434888729	0.0266	97.54	92.5	50.05
WT_24h_3	49840398	7395201495	0.0261	97.69	92.91	50.33
